# Using AI-Based Technologies to Help Nurses Detect Behavioral Disorders: Narrative Literature Review

**DOI:** 10.2196/54496

**Published:** 2024-05-28

**Authors:** Sofia Fernandes, Armin von Gunten, Henk Verloo

**Affiliations:** 1 School of Health Sciences University of Applied Sciences and Arts Western Switzerland (HES-SO) Sion Switzerland; 2 Les Maisons de la Providence Nursing Home Le Châble Switzerland; 3 Faculty of Biology and Medicine Institute of Higher Education and Research in Healthcare University of Lausanne Lausanne Switzerland; 4 Service of Old Age Psychiatry Lausanne University Hospital and University of Lausanne Lausanne Switzerland

**Keywords:** artificial intelligence, behavioral and psychological symptoms of dementia, neuropsychiatric symptoms, early detection, management, narrative literature review

## Abstract

**Background:**

The behavioral and psychological symptoms of dementia (BPSD) are common among people with dementia and have multiple negative consequences. Artificial intelligence–based technologies (AITs) have the potential to help nurses in the early prodromal detection of BPSD. Despite significant recent interest in the topic and the increasing number of available appropriate devices, little information is available on using AITs to help nurses striving to detect BPSD early.

**Objective:**

The aim of this study is to identify the number and characteristics of existing publications on introducing AITs to support nursing interventions to detect and manage BPSD early.

**Methods:**

A literature review of publications in the PubMed database referring to AITs and dementia was conducted in September 2023. A detailed analysis sought to identify the characteristics of these publications. The results were reported using a narrative approach.

**Results:**

A total of 25 publications from 14 countries were identified, with most describing prospective observational studies. We identified three categories of publications on using AITs and they are (1) predicting behaviors and the stages and progression of dementia, (2) screening and assessing clinical symptoms, and (3) managing dementia and BPSD. Most of the publications referred to managing dementia and BPSD.

**Conclusions:**

Despite growing interest, most AITs currently in use are designed to support psychosocial approaches to treating and caring for existing clinical signs of BPSD. AITs thus remain undertested and underused for the early and real-time detection of BPSD. They could, nevertheless, provide nurses with accurate, reliable systems for assessing, monitoring, planning, and supporting safe therapeutic interventions.

## Introduction

Demographic aging is a worldwide phenomenon, with significant growth in the number of older adults expected in the coming decades [1]. The number of people aged 80 years or older is expected to reach 426 million by 2050, with a high prevalence of dementia and other mental health disorders [2]. According to the World Health Organization, more than 55 million people worldwide endure dementia, and around 10 million new cases are diagnosed yearly [3]. More than 90% of them are affected by 1 or more of the behavioral and psychological symptoms of dementia (BPSD) and 80%-90% live in nursing homes. The BPSD, also known as neuropsychiatric symptoms related to dementia, is characterized by changes in behavior, perception, thought content, and mood [4]. The most common symptoms are apathy, aberrant motor behaviors, mood disturbances, aggression, anxiety, irritability, and sleep disorders [4]. The BPSD has a negative impact on the quality of life, accelerating functional decline and leading to earlier mortality [5,6]. The BPSD can be the source of social isolation, abuse, and burdens for informal caregivers [7,8]. For health care professionals, including nurses, managing BPSD can lead to work overload, stress, burnout, reduced quality of care, and the risks of patient abuse [9-11]. Finally, BPSD can increase health care system costs through more consultations, hospitalizations, and the prescription of more psychotropic drugs and mood stabilizers [12].

The etiopathogenesis of the BPSD is complex. Although dementia is a prerequisite for the onset of its behavioral and psychological symptoms, it is not the sole determinant. The BPSD can result from a convergence of factors, including neurological alterations, somatic problems, psychological factors, environmental conditions, and individual patient characteristics [13]. Moreover, the frequency, intensity, and types of symptoms vary considerably from 1 person to another. Thus, effectively managing the BPSD requires a structured approach that identifies and acts on various trigger factors [4,13-15]. The literature suggests different models or approaches, but all agree on three distinct steps which are (1) assessing manifestations of the BPSD that the patients present with, (2) formulating a hypothesis to help understand them, and (3) designing 1 or more interventions targeting their trigger factors [4,13-15]. Interventions can be psychosocial, pharmacological, or a combination of both [4,13-15]. Traditionally, detecting symptoms, monitoring their evolution, and evaluating treatment efficacy are based on nursing observations documented using assessment scales (Neuropsychiatric Inventory and Cohen-Mansfield Agitation Inventory) or solely using written notes in the patient’s medical record [15,16]. However, this process may prove ineffective for the early detection of the signs of BPSD, and nurses may perceive it as a potential factor in work overload [12]. Indeed, the first 2 steps require the investment of health care professionals, informal caregivers, and other individuals, and the third is more complex due to the variability and multifactorial nature of the BPSD. The BPSD challenges nurses daily, often triggering crises that are extremely complex to manage. Responding effectively and efficiently to these clinical issues requires more intensive observation and specialized care, with a greater emphasis on the prodromal detection of warning signs. However, due to an aging workforce and difficult working conditions (eg, high levels of stress and burnout, job dissatisfaction, and low levels of retention), the health care sector is facing a shortage of nursing staff [17]. The International Council of Nurses estimates a need for 13 million extra nurses to fill the worldwide shortages in the profession [18]. This shows the limitations of current human resources–based strategies, with the corollary need to explore innovative and sustainable solutions.

In recent decades, new information technologies have been adopted by every area of health care [19]. The first information technologies to be integrated into health care were electronic medical and health records, clinical information systems, and health information exchanges. More recently, other technologies have emerged, such as clinical decision support systems, mobile health apps, telehealth, telemedicine, robotics, wireless medical devices, and virtual reality [19-21]. Technological development in the health care sector, including nursing, is currently focused on artificial intelligence (AI) [19]. In the field of health care, AI usually refers to software capable of interpreting clinical data, learning from it, and helping clinical decision-making [19,22]. Combined with critical thinking and human judgment, AI has the potential to improve nurses’ clinical reasoning by increasing the speed and accuracy of assessment, anticipation, synthesis, and knowledge generation [23]. From 1985 to date, the PubMed database lists 1086 publications on AI in nursing. There has been a significant growth in the number of these publications since 2020, reinforcing the nursing sciences’ aims of developing and adapting nursing practices in line with sociodemographic changes and health care system, and medical and technical progress. [24]. Promoting the development, adoption and effective use of AI-based technologies (AITs) in health care has been identified as a key strategy to address the challenges related to both the complexity of managing the BPSD and limited resources [19,25-28]. Despite significant recent interest in the topic and the increasing number of technical devices on the market, little information is available on introducing AITs to help nurses attempting to detect BPSD as early as possible. This narrative review aims to identify and summarize the characteristics of existing publications concerning the use of AITs to support nurses in the early identification and management of BPSD.

## Methods

### Search Strategy

This narrative review was conducted following the Toronto and Remington guidelines [29]. The research question used to guide it was as follows:

What are the available publications on the use of artificial-intelligence-based technologies in neuropsychiatric symptoms related to dementia?

We consulted the PubMed database in September 2023 using the descriptors and keywords “artificial intelligence,” “behavioural and psychological symptoms of dementia,” and “neuropsychiatric symptoms” (Multimedia Appendix 1).

### Eligibility Criteria

Publications addressing the concept of dementia were included because the literature often links the concepts of dementia and the BPSD. The inclusion and exclusion criteria are presented in Textbox 1.

A total of 30 publications were identified and included after their titles and abstracts were reviewed. Following a thorough examination of their full texts, 5 publications were excluded because they focused on mental health issues unrelated to dementia or BPSD. In total, 25 publications were included.

**Inclusion and exclusion criteria.**

**Inclusion criteria**

- Mental health disorder
  Dementia
  Behavioral and psychological symptoms of dementia
- Health care setting
  No restrictions
- Artificial intelligence–based technology type
  No restrictions
- Artificial intelligence–based technology use
  No restrictions
- Publication type
  No restrictions
- Publication date
  No restrictions
- Language
  No restrictions

**Exclusion criteria**

- Mental health disorder
  Other mental health disorders

### Data Extraction and Synthesis

Information extracted from the publications retained for analysis included study design, country, journal title and category, mental health disorder addressed, type, subtype, use of the AITs, and health care setting. The type, subtype, and use of each AIT were identified via a basic qualitative content analysis, based on the authors’ stated aims and objectives (information found in the introduction and methods sections of the papers retained). In the context of mental health care, the type of AIT used was categorized according to the groups proposed by Jin et al [30], which are, machine learning, natural language processing, and digital health. Once this information was extracted, keywords were chosen to categorize the AIT’s use, with keywords determined based on the verbs used in each paper’s objectives (eg, measure, evaluate, screen, manage, and predict). The results are reported using a narrative approach.

## Results

### Study Characteristics

**Figure 1:**
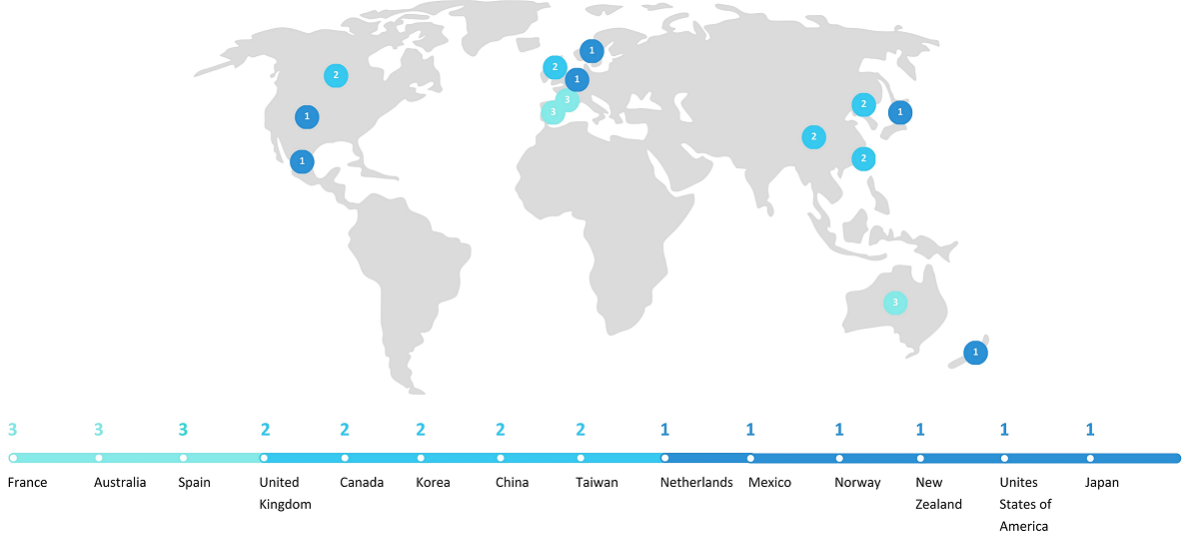
Number of publications per country.

Publications from 14 countries were identified, with publication dates ranging from 2006 to 2023 (Figure 1). A total of 8 publications addressed acute care settings [31-38], 6 looked at nursing homes [39-44], and 3 examined community care [45-47]. The majority described prospective observational studies published in journals covering geriatrics and psychogeriatrics [31,33,34,36,46-48] (Multimedia Appendix 2) [31-55].

### Uses of AITs

**Figure 2:**
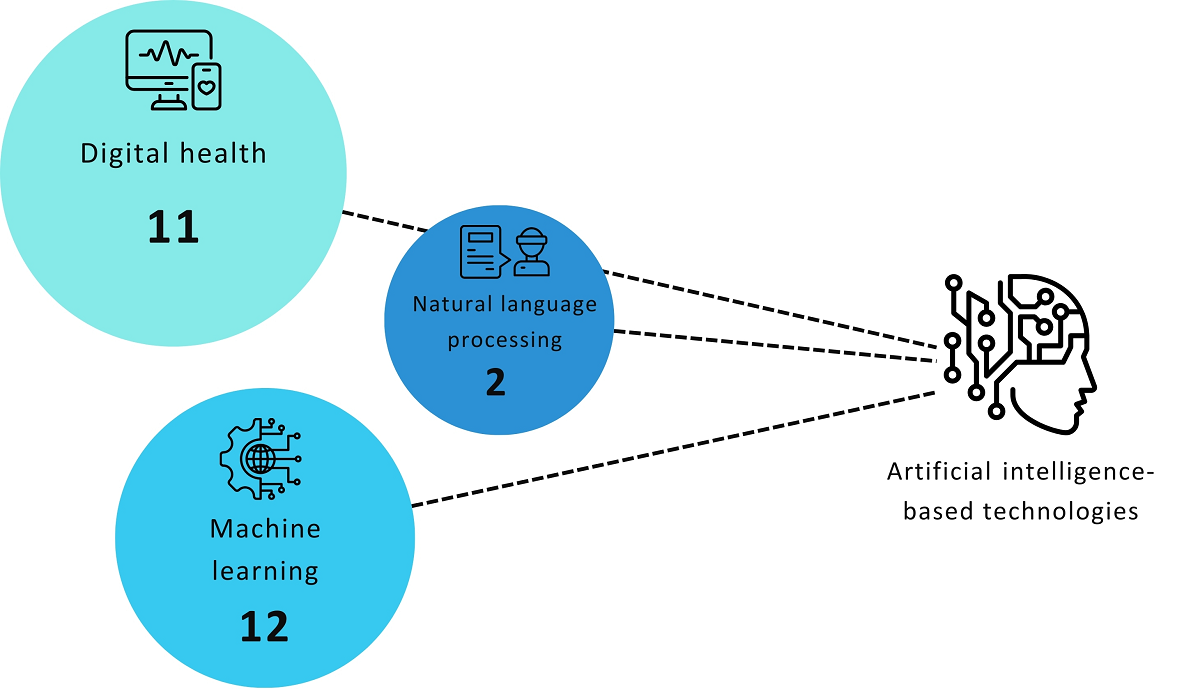
Number of publications by type of artificial intelligence–based technology.

A total of 12 publications reported using machine learning–type AITs, including the facial expression recognition and predictive modeling subtypes [31,34-38,43,46,47,49,50,55], 11 publications explored digital health–type AITs, including the wearable technologies and robotic subtypes [32,39-42,44,45, 51-54], and 2 publications examined natural language processing–type AITs [33,48] (Figure 2).

**Figure 3:**
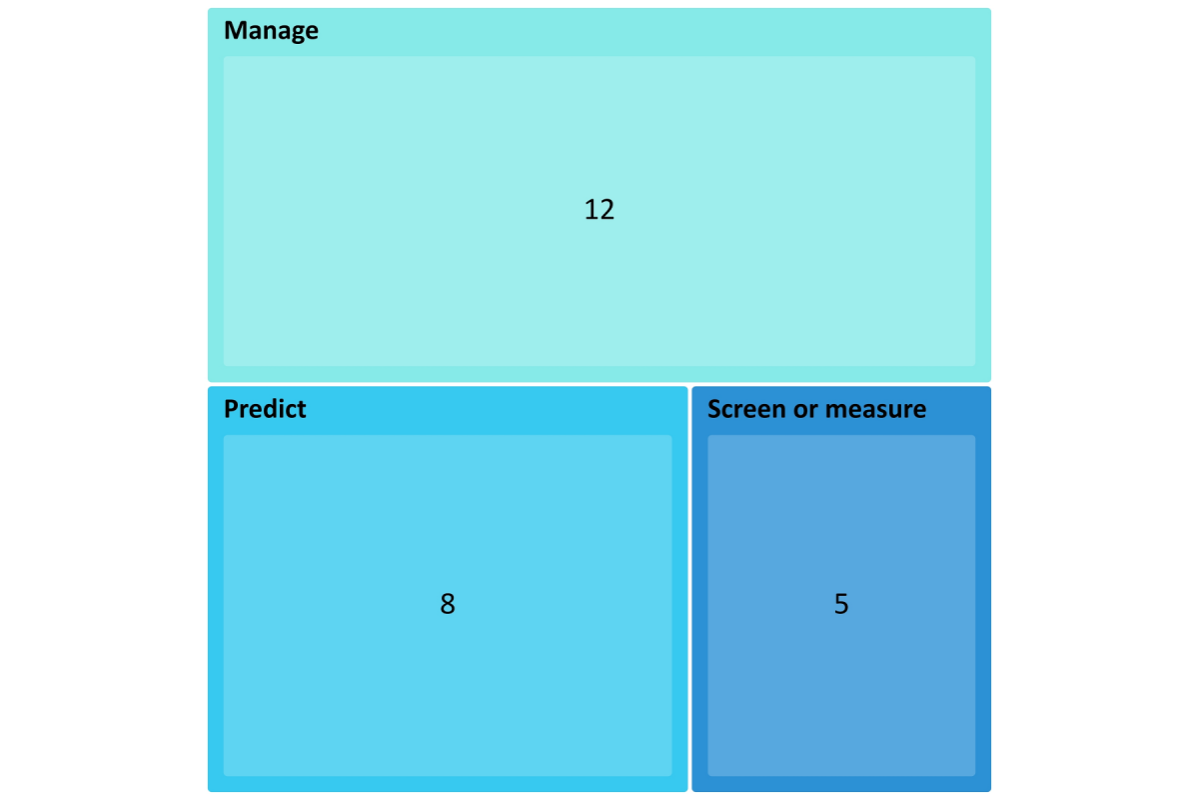
Number of publications by artificial intelligence–based technology use.

We identified three categories of publications depending on the AIT’s use and they are (1) predicting behavior and the stage and progression of dementia, (2) screening and assessing clinical symptoms, and (3) managing dementia and the BPSD (Figure 3).

A total of 4 publications reported on the use of machine learning technology to predict dementia behavior and the stage and progression of dementia [34,36-38]. Three publications referred to the use of natural language processing for screening and assessing clinical symptoms [35,48,49] and 1 reported on the use of machine learning [50]. One publication described the use of wearable technologies [40], and 1 combined this type of AIT with machine learning [46]. Finally, 10 publications reported on the use of robotics as a psychosocial approach to managing dementia and BPSD [32,39,41,42,44,45,51-54] (Table 1).

**Figure 4:**
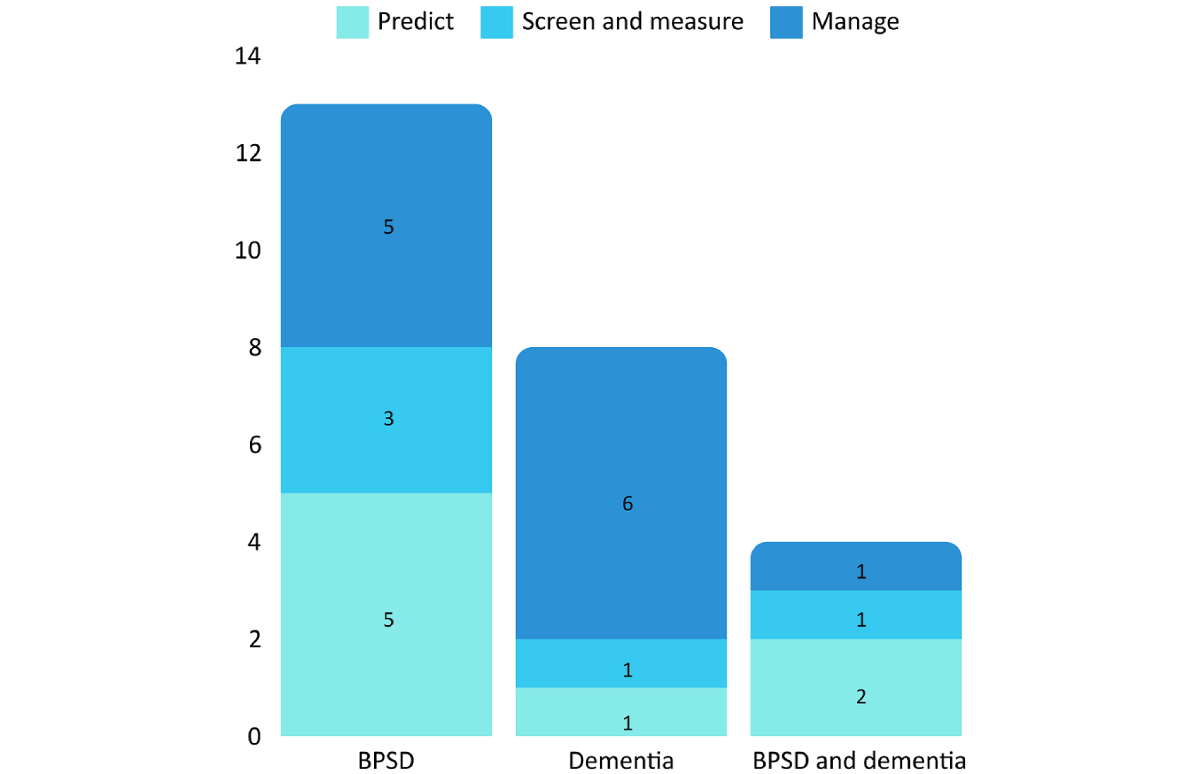
Number of publications by artificial intelligence–based technology use and mental health issues. BPSD: behavioral and psychological symptoms of dementia.

One publication reported on the use of AITs to predict the stage and progression of dementia [31], 1 described the detection and measurement of dementia’s clinical symptoms [48], and 6 examined dementia management [39,41,44,51-53]. As for the BPSD, 5 publications reported using AITs to predict behavior [33,36,37,46,47], 3 described the detection and assessment of clinical symptoms [35,40,49], and 5 looked at managing behavior [32,42-44,55] (Figure 4). In the context of the BPSD, behavior management refers to interventions carried out to identify and act on trigger factors.

**Table 1:** Publications by type of AIT^a^ and use.

Reference	AIT types			AITs subtype		AITs use		
	Machine learning	Natural language processing	Digital health			Predict	Screen and measure	Manage
Al-Harrasi et al [48]		✓			N/A^b^		✓	
Byeon [31]	✓				Predictive modeling	✓		
Chen et al [39]			✓		Robotics			✓
Chen et al [47]	✓				Facial expression recognition	✓		
Cho et al [46]	✓				Predictive modeling	✓		
Demange et al [32]			✓		Robotics			✓
Eikelboom et al [33]		✓			N/A	✓		
Favela et al [40]			✓		Wearable technology		✓	
Filan and Llewellyn-Jones [51]			✓		Robotics			✓
Gill et al [34]	✓				N/A	✓		
Hsieh et al [41]			✓		Robotics			✓
Jøranson et al [44]			✓		Robotics			✓
König et al [49]	✓				N/A		✓	
König et al [35]	✓				N/A		✓	
Leng et al [52]			✓		Robotics			✓
Liang et al [45]			✓		Robotics			✓
Mallo et al [36]	✓				N/A	✓		
Mar et al [38]	✓				N/A	✓		
Mar et al [37]	✓				N/A	✓		
Moyle et al [42]			✓		Robotics			✓
Pu et al [53]			✓		Robotics			✓
Russo et al [55]	✓				N/A			✓
Shah et al [50]	✓				N/A		✓	
Tadokoro et al [43]	✓				Facial expression recognition			✓
Yu et al [54]			✓		Robotics			✓

^a^AIT: artificial intelligence–based technology.

^b^N/A: not applicable.

## Discussion

### Principal Results

Despite the growing interest in AITs, most of those currently used take a psychosocial approach to treating and caring for patients with BPSD by using the clinical signs that are already present. AI remains largely unexplored in terms of its potential for the early, real-time detection of BPSD. Yet, in different health care settings and contexts, AITs could provide nurses with accurate and reliable systems for assessing, monitoring, planning, and supporting safe therapeutic interventions [27,56]. Based on our findings, it appears that the use of AITs has been explored more in acute care than in long-term care settings, which include community care and nursing homes. However, the prevalence of BPSD seems to be higher in the context of long-term care, particularly in nursing homes where 80%-90% of residents exhibit at least 1 of the BPSD and institutional resources tend to be more limited [57].

As mentioned above, BPSD has traditionally been assessed and monitored by health care professionals’ observations of patients’ behaviors [15,16]. However, this process may have limited success in the early detection of warning signs of the BPSD, and health care professionals perceive it to be another task or factor leading to work overload [11]. Therefore, it seems appropriate to anticipate symptom escalation and optimize staff and financial resources. Multimodal sensors for capturing physiological parameters, activity trackers, and facial expression recognition are all promising AITs that make the process of managing the BPSD more efficient and personalized [40,58-61]. By mining information from such devices, nurses could detect early warning signs of BPSD and their trigger factors. By combining this information with their clinical knowledge and experience, nurses could be equipped with a clinical decision-making support system enabling them to guide and personalize their therapeutic interventions [56,62-64].

Although nurses agree about the potential usefulness of AITs, most do not fully understand AI’s underlying principles, and they are concerned about the potential consequences of its use in clinical practice [65-68]. Other obstacles pertaining to AITs include the unknown cost-benefits of their use in health care settings, the current lack of use and data management protocols in those settings, and the lack of information technology capacity there to support them [69,70]. The published papers identified in this review reinforced these points as the involvement of nurses in designing studies and the use of AITs was low. However, in interdisciplinary contexts, nurses have key roles to play in the conception and design of AIT devices, verifying their effectiveness and adapting their use.

### Strengths and Limitations

The characteristics of the publications retained in this narrative review revealed the countries and contexts where AITs have been integrated into settings dealing with the BPSD and have been investigated. It also demonstrated the types of technologies available and their intended purposes, as well as the clinical contexts in which they are deployed. These results, while not exhaustive, provide a preliminary overview of this emerging topic and identify AITs’ potential benefits for clinical practice and pathways for future research. This narrative review has some limitations, nevertheless. The absence of an assessment of the quality and validity of the selected publications may bias the quality of their reported outcomes. Furthermore, including publications that address dementia could lead to confusion regarding this narrative review’s focus. Although the concepts of dementia and BPSD are frequently interrelated in the specialized literature, including the concept of dementia could lead readers to misunderstand the scope of the results presented.

### Conclusions

AI has the potential to transform nursing practice, particularly in support of the diagnosis and management of BPSD, which are currently among the major challenges in caring for older adults with dementia. However, our literature review found little experimental evidence, data, or understanding of how these types of technologies could be applied advantageously to the early detection of BPSD by nurses. Furthermore, although these are preliminary findings, the results of this review showed that research on this topic has only been done in relatively few countries, despite the impact of the BPSD being a global phenomenon. Based on this fact and the review’s limitations, we would recommend that a more comprehensive examination be performed, such as a scoping review, to meticulously explore the research conducted on AITs for the early detection of BPSD. It also seems important that future experimental research investigates the effectiveness, feasibility, and acceptability of using devices based on AITs for the prodromal detection of BPSD. Specific research in long-term care settings seems to be particularly lacking. Nurses are intimately involved in creating a vision of contemporary professional nursing practice and then applying that practice. Therefore, it seems appropriate that they should be involved in strengthening collaboration with information technology engineers and programmers. Nurses’ perceptions and experiences of using AITs to detect BPSD should also be explored, using a qualitative approach, as should how the data provided by these types of technologies contribute to nurses’ clinical reasoning and decision-making processes.

## Appendix

Multimedia Appendix 1 Search query.

Multimedia Appendix 2 Description of the selected publications.

## Abbreviations

AI: artificial intelligence
AIT: artificial intelligence–based technology
BPSD: behavioral and psychological symptoms of dementia
