# Supplementary material for: Using AI-Based Technologies to Help Nurses Detect Behavioral Disorders: Narrative Literature Review
Source: JMIR Nurs. 2024 May 28;7:e54496. doi: 10.2196/54496 (PMC11167323; doi:10.2196/54496)
Supplement: Multimedia Appendix 1 [file nursing_v7i1e54496_app1.docx]

| **Database : PubMed** | |
| --- | --- |
| **Search query** | **Results** |
| **AI and BPSD** | |
| ("Artificial Intelligence"[MeSH Terms] OR "artificial intelligence"[Title/Abstract]) AND ("behavioral and psychological symptoms of dementia"[Title/Abstract] OR "neuropsychiatric symptoms"[Title/Abstract]) | 30 |
