# Supplementary material for: Using AI-Based Technologies to Help Nurses Detect Behavioral Disorders: Narrative Literature Review
Source: JMIR Nurs. 2024 May 28;7:e54496. doi: 10.2196/54496 (PMC11167323; doi:10.2196/54496)
Supplement: Multimedia Appendix 2 [file nursing_v7i1e54496_app2.docx]

| Authors | Publication year | Country | Study design | Healthcare setting | Journal title | Journal category |
| --- | --- | --- | --- | --- | --- | --- |
| Al-Harrasi et al [48] | 2021 | United Kingdom | Prospective observational | N/A | Experimental Gerontology | Biogerontology |
| Byeon [31] | 2021 | Korea | Prospective observational | Hospital | International Journal of Environmental Research and Public Health | Environmental, public and occupational health |
| Chen et al [39] | 2020 | China | Multicentre randomised controlled trial | Nursing home | Journal of the American Medical Directors Association | Post-acute and long-term care medicine |
| Chen et al [47] | 2022 | Taiwan | Prospective observational | Daycare centre | Aging | Ageing |
| Cho et al [46] | 2023 | Korea | Prospective observational | Home care | Scientific Reports | Natural sciences |
| Demange et al [32] | 2018 | France | Single-centre, pilot-controlled trial | Hospital | Clinical Interventions in Aging | Gerontology |
| Eikelboom et al [33] | 2023 | Netherlands | Prospective observational | Hospital | Alzheimer's Research & Therapy | Alzheimer’s disease |
| Favela et al [40] | 2020 | Mexico | Mixed methods (QUANT 🡪 QUAL) | Nursing home | Journal of Biomedical Informatics | Health informatics, translational bioinformatics |
| Filan and Llewellyn-Jones [51] | 2006 | Australia | Literature review of controlled trials | N/A | International Psychogeriatrics | Psychogeriatrics |
| Gill et al [34] | 2020 | Canada | Prospective observational | Hospital | Journal of Alzheimer’s Disease | Alzheimer’s disease |
| Hsieh et al [41] | 2023 | Taiwan | Systematic review and meta-analysis | Nursing home | Gerontology | Geriatrics, gerontology |
| Jøranson et al [44] | 2015 | Norway | Multicentre, cluster-randomised controlled trial | Nursing home | Journal of the American Medical Directors Association | Post-acute and long-term care medicine |
| König et al [49] | 2019 | France | Single-centre controlled trial | N/A | Journal of Alzheimer’s Disease | Alzheimer’s disease |
| König et al [35] | 2021 | France | Prospective interventional | Hospital | European Psychiatry | Psychiatry |
| Leng et al [52] | 2019 | China | Systematic review and meta-analysis | N/A | Psychiatry Research | Psychiatry |
| Liang et al [45] | 2017 | New Zealand | Pilot multicentre, randomised controlled trial | Home care | Journal of the American Medical Directors Association | Post-acute and long-term care medicine |
| Mallo et al [36] | 2020 | Spain | Prospective observational | Hospital | International Psychogeriatrics | Psychogeriatrics |
| Mar et al [38] | 2022 | Spain | Retrospective | Hospital | *Revista de Psiquiatría y Salud Mental* | Psychiatry and mental health |
| Mar et al [37] | 2020 | Spain | Retrospective | Hospital | Journal of Alzheimer’s Disease | Alzheimer’s disease |
| Moyle et al [42] | 2019 | Australia | Descriptive qualitative study | Nursing home | The Gerontologist | Ageing |
| Pu et al [53] | 2022 | Australia | Protocol for feasibility of a single-centre randomised controlled trial | Nursing home | Journal of Advanced Nursing | Nursing |
| Russo et al [55] | 2023 | Canada | Perspective | N/A | Frontiers in Digital Health | Health informatics, health technology innovation |
| Shah et al [50] | 2023 | United States of America | Literature review | N/A | Journal of Alzheimer’s Disease | Alzheimer’s disease |
| Tadokoro et al [43] | 2021 | Japan | Single-centre controlled trial | Nursing home | Journal of Alzheimer’s Disease | Alzheimer’s disease |
| Yu et al [54] | 2022 | United Kingdom | Systematic review and meta-analysis | N/A | Ageing Research Reviews | Ageing |
